# Notes

**Published:** 1901-05

**Authors:** 


### NOTES.

Dr. R. B. Plageman, of Brooklyn, N. Y., takes a keen interest in all matters pertaining to canine pathology, and is the author of *House Dogs; their Care and Treatment.*

Veterinarian J. H. Pear, of Saugatuck, Mich., has just been reëlected Mayor of that city. Dr. Pear has always taken an active interest in municipal affairs, and served as alderman prior to his elevation to the mayoralty.

Governor Nash, of Ohio, has just appointed Dr. A. E. Cunningham, of Cleveland, to succeed Dr. A. Logan, of Bellefontaine, and Dr. Walter Shaw, of Dayton, to succeed Dr. N. B. Smith, of Fairfield County, as members of the State Veterinary Board of Examiners. Dr. A. E. Cunningham is a graduate of the Veterinary Department of the University of Pennsylvania and Dr. Walter Shaw of the Ontario Veterinary College.

Dr. I. White, formerly of Manhattan, N. Y., is now located in New York City.

Dr. Louis Metsker, formerly engaged in the Bureau of Animal Industry work at Kansas City, Mo., is now located at Albuquerque, N. M.

Dr. J. J. Hougendobler, of Mountville, Pa., has removed to Harrisburg, of the same State.

